# Couple-level determinants of syphilis infection among heterosexual married couples of reproductive age in Guangdong Province, China: A population-based cross-sectional study

**DOI:** 10.3389/fpubh.2022.1004246

**Published:** 2022-10-17

**Authors:** Lu Han, Wenxue Xiong, Mingzhen Li, Rui Li, Jiabao Wu, Xijia Tang, Li Ling, Xiaohua Liu

**Affiliations:** ^1^National Health Commission (NHC) Key Laboratory of Male Reproduction and Genetics, Guangdong Provincial Reproductive Science Institute (Guangdong Provincial Fertility Hospital), Guangzhou, China; ^2^Faculty of Medical Statistics, School of Public Health, Sun Yat-sen University, Guangzhou, China; ^3^Clinical Research Design Division, Clinical Research Center, Sun Yat-sen Memorial Hospital, Sun Yat-sen University, Guangzhou, China

**Keywords:** syphilis, determinants, heterosexual couples, married couples, couple-level, China

## Abstract

**Background:**

Syphilis remains a major public health problem worldwide, and its prevention requires knowledge of factors that go beyond the individual-level. However, most syphilis-related studies have focused on individual-level and regional-level factors, neglecting couple-level factors. Thus, this study aimed to explore couple-level determinants of syphilis infection among heterosexual married couples.

**Methods:**

This population-based cross-sectional study used data from heterosexual married couples who participated in the National Free Preconception Health Examination Project in Guangdong Province, China during 2014–2019. The syphilis infection was tested by the rapid plasma reagin test. Couple-level data were obtained by combining information provided by the man and woman living in the same household. Multivariate logistic models were employed to explore the couple-level determinants of syphilis infection by gender after adjusting for potential confounders.

**Results:**

A total of 1,755,156 couples were recruited in this analysis. The seroprevalence was 0.25% (95%CI: 0.24–0.25%) and 0.26% (95%CI: 0.25–0.27%) among men and women, respectively. The median age was 28.0 (interquartile range, IQR: 25.0–31.0) years, and the median duration of marriage was 0.2 (IQR: 0.0–2.5) years. After adjusting for individual and regional-level variables, duration of marriage was a protective factor for syphilis infection in men (adjusted odds ratios, AOR: 0.97; 95% CI: 0.96–0.98) and women (AOR: 0.95, 95% CI: 0.94–0.96). The age gap and the difference in education level between the husband and wife were associated with syphilis infection, but these associations were somewhat different between men and women. Condom use was negatively associated with syphilis infection in men (AOR: 0.77; 95% CI: 0.70–0.84) and women (AOR: 0.77, 95% CI: 0.71–0.84). Our results also showed that couple mobility and the number of children were not statistically significant determinants of syphilis infection among heterosexual married couples.

**Conclusion:**

This study contributes to a more comprehensive understanding of syphilis outcomes in individuals in the context of marriage in China. Several couple-level factors are indeed associated with syphilis infection, but these associations differ between men and women. Couple-based strategies that engage both women and men and efforts to promote condom use among heterosexual married couples need to be developed and further evaluated for syphilis prevention.

## Introduction

Around the world, syphilis continues to be a major public health issue (1), with ~6 million new cases reported annually (2) and an estimated 1,07,000 fatalities (3). Patients also experience a great deal of stigma (4, 5). In China, where syphilis has been endemic again since the 1980s (6), the incidence of syphilis has increased more than any other mandatorily reported infectious disease (7). According to earlier research (8), people of reproductive age (21–49 years old) had the highest incidence. Additionally, syphilis infection in either the wife or the husband could lead to congenital syphilis (CS) (9), the most common congenital infection worldwide, which has tremendous consequences for the mother and her fetus (10, 11).

Epidemiologic studies of the spread of infectious diseases have increasingly recognized the importance of factors that go beyond the individual level (12, 13). In particular, the transmission of sexually transmitted infections (STIs) like syphilis often involves interactions that occur in relationship settings (14). Therefore, analyses targeting individual-level factors alone may not adequately identify interpersonal drivers of syphilis transmission (15).

Marriage is one of the most influential social relationships and a growing body of research suggests that many couple-level factors may influence health status (16). For example, a study in Northern Tanzania showed that the duration of marriage and number of children were associated with HIV infection among women (17). Based on a long-term study of siblings and their spouses, it was reported that spousal education was positively associated with health (18). The age gap and difference in education level between the husband and wife may also affect maternal reproductive health (19). However, the associations between these couple-level factors and syphilis infection were not clear. A multilevel study in Shenzhen City, China also showed that migrants were more likely to contract syphilis infection (20). Moreover, unprotected sex within heterosexual married couples was common (15), which was a strong risk factor for syphilis infection. However, previous studies exploring determinants of syphilis infection have mostly focused on individual-level and regional-level factors (21, 22), neglecting couple-level factors.

In China, the National Free Preconception Health Examination Project (NFPHEP) was initiated by the National Health and Family Planning Commission of the People's Republic of China in 2010. This project provided free syphilis screening for married couples planning to be pregnant within the next 6 months and collected individual-level and couple-level data through a standardized questionnaire (23), providing an opportunity to fill the research gap mentioned above. Thus, this study aimed to explore couple-level determinants of syphilis infection among heterosexual married couples of reproductive age and provide a reference for public health decision makers.

## Materials and methods

### Study area

**Figure 1:**
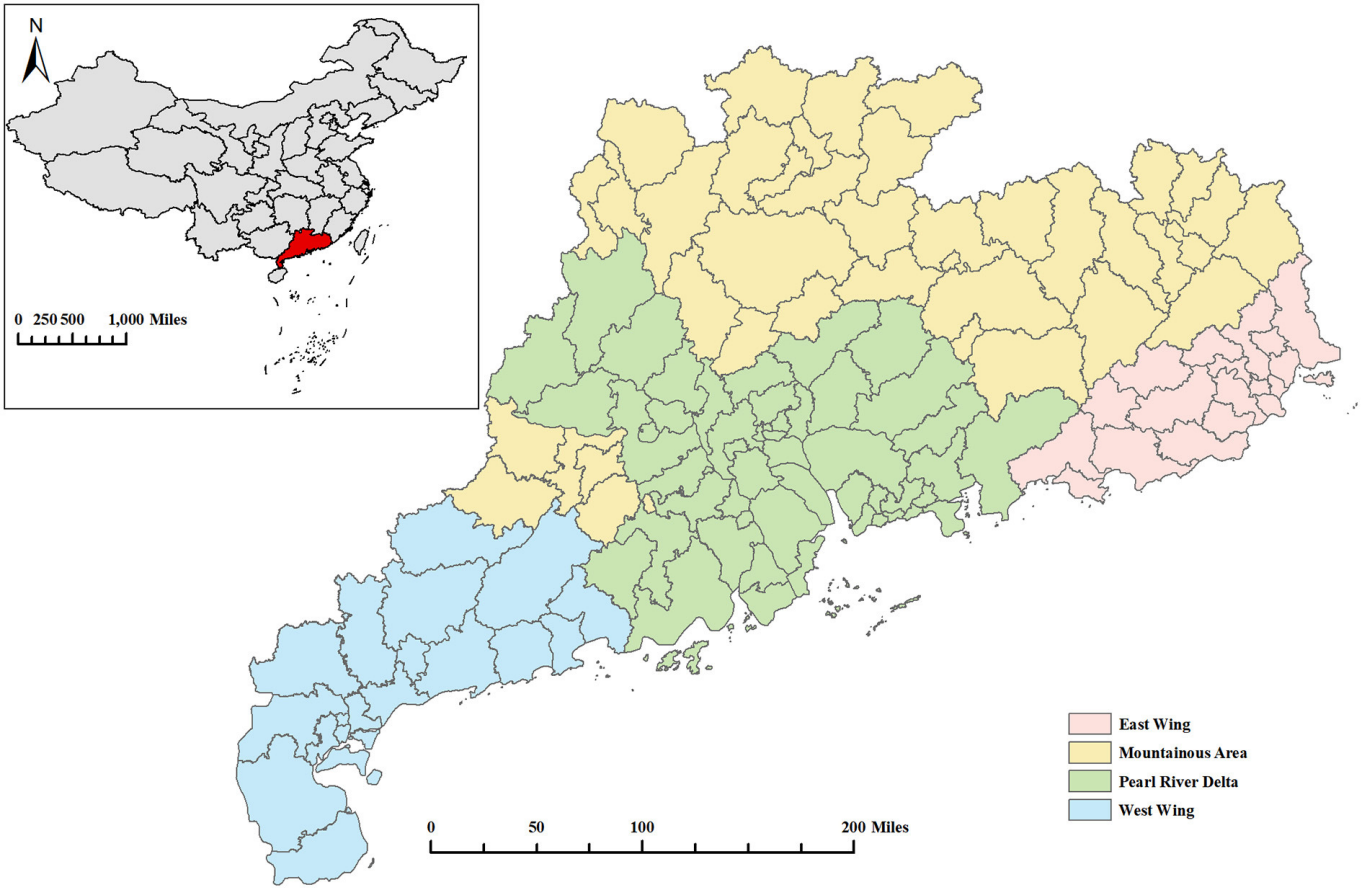
The location of Guangdong Province and the division of its four regions. Base layer of the map was downloaded from Resource and Environment Science and Data Center (https://www.resdc.cn/data.aspx?DATAID=201).

Guangdong Province, located in South China (Figure 1), is a microcosm of China, with very uneven economic development across regions and widely varying population densities. Specifically, Guangdong Province was divided into four regions: the Pearl River Delta, East Wing, West Wing, and Mountainous Area. The Pearl River Delta region accounted for 80% of the gross domestic product (GDP) and 56% of the total population of Guangdong, but it made up only 30% of the land of Guangdong (24). It was reported that in 2019, the resident population of Guangdong Province was 120 million (one ninth of the entire population of China), of which 41% were migrants and 22.4 million were married women of reproductive age (25). Like other provinces in China, Guangdong witnessed a rapid increase in reported syphilis cases from 55,936 in 2014 to 62,760 in 2019 (25). The NFPHEP was well-implemented in Guangdong Province, with a coverage rate of over 85% of the target population (25), providing us with sufficient data to investigate couple-level determinants of syphilis infection.

### Study design and participants

This population-based cross-sectional study used data from heterosexual married couples who participated in the NFPHEP in Guangdong Province, China, between January 2014 and November 2019. The NFPHEP provided free preconception health services for married couples in Guangdong Province who met the following criteria: (a) intending to conceive within the next 6 months; (b) at least one of them was a local resident, or neither was a local resident, but both had lived in locale for more than 6 months. Couples who are planning a pregnancy are volunteering to participate in the NFPHEP and sociodemographic characteristics of each spouse were obtained face-to-face by trained health workers using a standardized questionnaire separately. Various forms of social media, such as newspapers, television, and radio were used to make the targeted population aware of the project. The program has also been implemented by setting up counseling points at marriage registration offices.

Blood samples were collected by trained and qualified nurses. Then samples were then stored at 4–8°C and transported for analysis within 24 h. Syphilis serology testing was performed by the rapid plasma reagin (RPR) test in local laboratories affiliated to medical institutions under qualified quality control mechanisms. Test kits approved by National Medical Products Administration were selected by local laboratories according to their preferences. If the result of test was positive, the health workers would inform the person and their spouses, and refer them to a specialist for further confirmation and treatment. Quality control was performed through the following measures: (a) establish the commission of the National Center of Clinical Laboratories for Quality Inspection and Detection, including experts from chemical, clinical, microbiological, and immunological laboratories; (b) convene of committees to discuss critical factors that may affect laboratory results and draft quality control plans for key items; (c) conduct external quality assessments twice a year, covering 13 items (including the results of the RPR test) (26, 27). Each participant provided written informed consent prior to enrollment and the project was approved by the Institutional Review Board of the Chinese Association of Maternal and Child Health Studies (IRB-201001).

**Figure 2:**
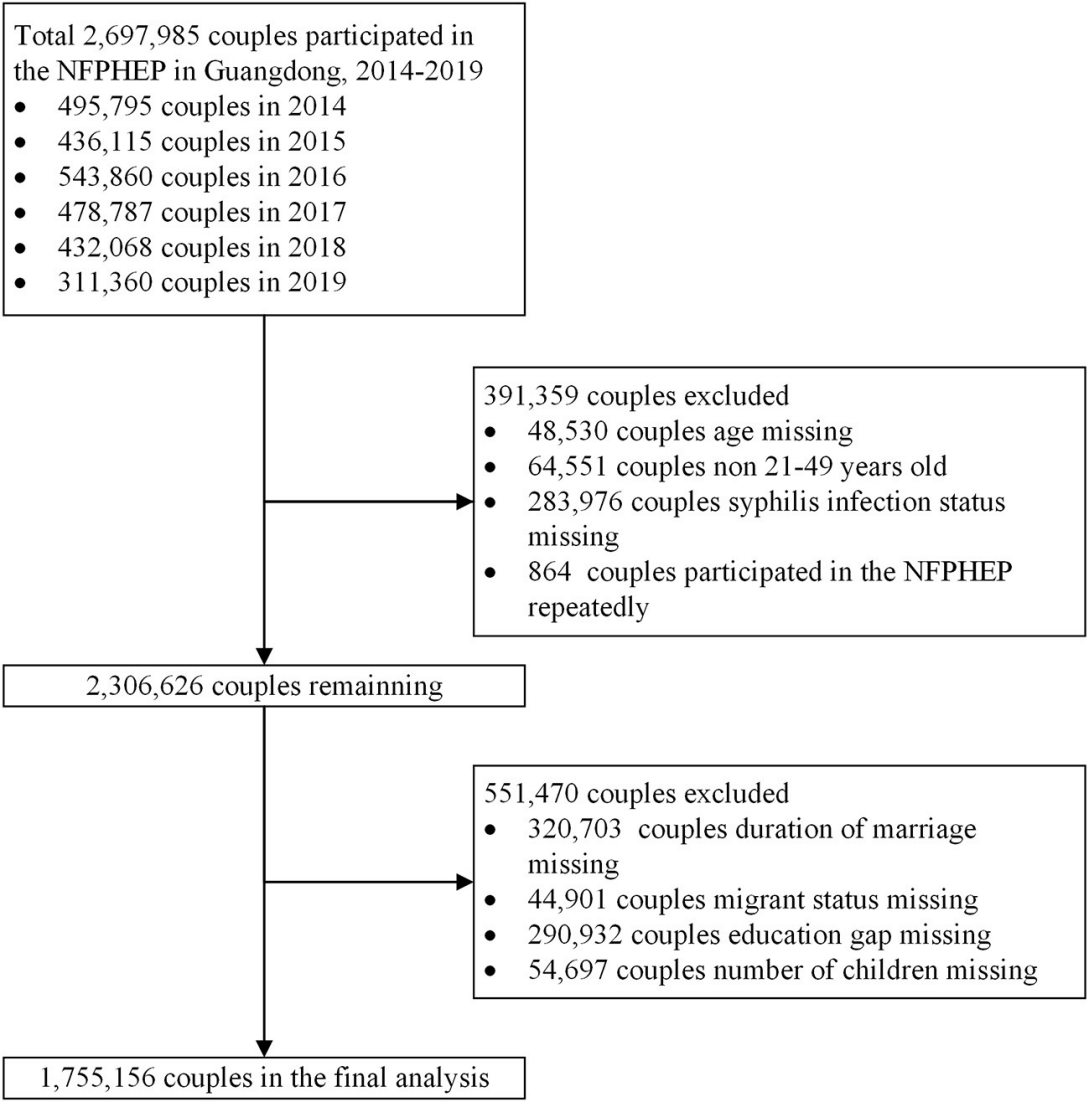
Flow chart of participants in the National Free Preconception Health Examination Project (NFPHEP) in Guangdong Province, China during 2014–2019.

We applied the commonly used definition of reproductive age (i.e., 21–49 years old) according to previous studies (22, 28). The inclusion criteria of this study were as follows: Couples who participated in the NFPHEP in Guangdong during 2014–2019; Couples in which both the wife and the husband were between 21 and 49 years of age; Couples with available syphilis serology tests. Couples who failed to complete the RPR test or participated in the project repeatedly were excluded. In addition, as this study aimed to explore couple-level determinants of syphilis infection, couples who lacked couple-level factors were excluded (Figure 2).

### Outcome and covariates

The dependent variable was syphilis infection, which was tested by the RPR test and dichotomized as positive and negative. The independent variables in this study included individual-level, couple-level, and regional-level factors, with individual-level and regional-level factors regarded as potential confounders (12, 22). The definitions of these factors are present in Supplementary Table 1. Individual-level and regional-level characteristics were as follows: year of screening (2014, 2015, 2016, 2017, 2018 or 2019); respondent's age (as continuous variable); ethnicity (Han or minority); respondent's educational level (primary school or below, middle and high school or college and above); household registration (rural or urban); smoking (no or yes); drinking (no or yes); illicit drug use (no or yes); previous STIs (no or yes) and region (Pearl River Delta, East Wing, West Wing, or Mountainous Area). Participants would be identified to be domestic migrants if they did not participant in the program in the household registered city, and vice versa. The household registration location was based on the HUKOU system in China (29), while the city in which they were enrolled was based on the administrative division code from the Ministry of Civil Affairs of the People's Republic of China (30). All information obtained was then uploaded to a specialized web-based reporting system that generates a unique number for each couple.

Couple-level data were obtained by combining information provided by the man and woman living in the same household. Duration of marriage was defined as the difference in time (in years) between participation in the NFPHEP and registration of the marriage. The definition of age gap could vary according to previous studies (19, 31). In this study, we calculated the age gap within couples and arbitrarily categorized them as follows: no age gap, wife older than husband, husband 1–2 years older, husband 3–5 years older, or husband ≥6 years older. We also classified couples as local couples (both the wife and husband were native residents) or migrant couples (either the wife or husband or both were migrant). Condom use: no or yes. Difference in education level between the husband and wife: same education level, husband had a higher education, or wife had a higher education. The number of children: 0 or ≥1.

### Statistical analysis

We used the median and interquartile range (IQR) to describe quantitative variables with a skewed distribution. The categorical variables were described by numbers and percentages and tested using Chi-square tests. The Cramer's V was calculated in Chi-squared tests to estimate effect sizes (32). General logistic regression stratified by gender, rather than the multilevel model (14), was employed to estimate the associations between couple-level factors and syphilis infection for three reasons. First, it is difficult to make the multilevel model converge due to the extremely large samples. Second, syphilis infection was highly correlated between the husband and wife, which did not satisfy the basic assumption of independence, so the general logistic regression was stratified by gender. Third, the associations between couple-level factors and syphilis infection in men and women could be explored separately.

All variables were tested for multicollinearity in a preliminary ordinary least square regression analysis. We used the variance inflation factor (VIF) <5 to control the potential multicollinearity problem. We first conducted univariate logistic regression models to calculate the crude odds ratios (COR) of couple-level factors. Then the multivariate logistic model was employed to estimate the associations between couple-level factors and syphilis infection. Three models were fitted, where Model A included only couple-level factors, Model B additionally adjusted for individual-level factors, and Model C additionally adjusted for individual-level and regional-level factors. Complete cases were used in the above models. The results of associations were presented as adjusted odds ratios (AOR) with corresponding 95% confidence intervals (CI) and the description of the final results was primarily focused on Model C.

We also performed a stratified analysis to investigate whether the associations varied across regions. In addition, we created a “Not available” category for those with missing data at the individual-level to perform a robustness check. We considered a two-sided test *P* < 0.05 to be statistical significance. All analyses were performed with R version 4.2.1.

## Results

### Characteristics of the participants

The overall seroprevalence of syphilis was 0.25% among 3,510,312 participants from 1,755,156 couples (Table 1). The seroprevalence was 0.25% (95%CI: 0.24–0.25%) and 0.26% (95%CI: 0.25–0.27%) among men and women, respectively. The median age of all participants was 28 (IQR: 25.0–31.0) years, 71.5% were between the ages of 21 and 30, 97.1% were of Han ethnicity, 58.2% had middle and high school education, and 17.6% were migrants. Among 1,755,156 couples, the median duration of marriage was 0.2 (IQR: 0.0–2.5) years, 68.0% of the husband was older than the wife, 71.5% were local couples (husband and wife were both local residents), 75.3% did not use condoms, 68.7% had no children, and 68.6% had the same education level (Table 2). Distribution of syphilis infection of participants stratified by gender was shown in Supplementary Table 2.

**Table 1:** Individual-level factors of heterosexual married individuals who participated in National Free Preconception Health Examination Project in Guangdong Province, China during 2014–2019 (*N* = 3,510,312).

**Characteristic**	**Total number (%)**	**Cases (%)**	**Seroprevalence** **(%), 95%CI**	**Cramer's V**	***P*-value^#^**
**Total**	3,510,312 (100.0)	8,921 (100.0)	0.25 (0.25–0.26)		
**Year of screening**
2014	651,720 (18.6)	1,782 (20.0)	0.27 (0.26–0.29)	0.004	<0.001
2015	580,288 (16.5)	1,464 (16.4)	0.25 (0.24–0.27)		
2016	700,268 (19.9)	1,968 (22.1)	0.28 (0.27–0.29)		
2017	625,640 (17.8)	1,545 (17.3)	0.25 (0.23–0.26)		
2018	540,660 (15.4)	1,242 (13.9)	0.23 (0.22–0.24)		
2019	411,736 (11.7)	920 (10.3)	0.22 (0.21–0.24)		
**Gender**
Man	1,755,156 (50.0)	4,350 (48.8)	0.25 (0.24–0.26)	0.001	0.020
Woman	1,755,156 (50.0)	4,571 (51.2)	0.26 (0.25–0.27)		
Age*			28.0 (IQR: 25.0–31.0)		
**Ethnicity** ^**##**^
Han	3,473,690 (99.1)	8,793 (98.7)	0.25 (0.25–0.26)	0.002	<0.001
Minority	29,892 (0.9)	114 (1.3)	0.38 (0.31–0.46)		
**Educational level**
Primary school or below	75,718 (2.2)	405 (4.5)	0.53 (0.48–0.59)	0.056	<0.001
Middle and high school	2,044,394 (58.2)	6,437 (72.2)	0.31 (0.31–0.32)		
College and above	1,390,200 (39.6)	2,079 (23.3)	0.15 (0.14–0.16)		
**Household registration** ^**##**^
Rural	2,541,175 (72.5)	6,918 (77.6)	0.27 (0.27–0.28)	0.006	<0.001
Urban	965,586 (27.5)	1,998 (22.4)	0.21 (0.20–0.22)		
**Smoking** ^**##**^
No	3,011,751 (86.1)	7,159 (80.6)	0.24 (0.23–0.24)	0.008	<0.001
Yes	484,976 (13.9)	1,724 (19.4)	0.36 (0.34–0.37)		
**Drinking** ^**##**^
No	2,699,543 (77.3)	76,733 (75.8)	0.25 (0.24–0.26)	0.002	0.001
Yes	794,586 (22.7)	2,146 (24.2)	0.27 (0.26–0.28)		
**Migrant**
No	2,893,109 (82.4)	7,256 (81.3)	0.25 (0.24–0.26)	0.001	0.008
Yes	617,203 (17.6)	1,665 (18.7)	0.27 (0.26–0.28)		
**Previous STIs**
No	3,444,225 (98.1)	8,512 (95.4)	0.25 (0.24–0.25)	0.010	<0.001
Yes	66,087 (1.9)	409 (4.6)	0.62 (0.56–0.68)		
**Illicit drug use** ^**##**^
No	3,486,866 (100.0)	8,846 (99.9)	0.25 (0.25–0.26)	0.002	<0.001
Yes	521 (0.0)	7 (0.1)	1.34 (0.54–2.75)		

Data was presented as No. (%); reported percentages are composition ratios of each horizontal item.

^#^The seroprevalence in subgroups was tested by Chi-square test.

*Data was described by median and interquartile range (IQR).

^##^6,730 (0.2%) missing in ethnicity; 3,551 (0.1%) missing in household registration; 13,585 (0.4%) missing in smoking; 16,183 (0.5%) missing in drinking; 23,826 (0.6%) missing in drug use.

**Table 2:** Couple-level and regional-level factors of heterosexual married individuals who participated in National Free Preconception Health Examination Project in Guangdong Province, China during 2014–2019 (*N* = 3,510,312).

**Characteristic**	**Total number (%)**	**Cases (%)**	**Seroprevalence** **(%), 95%CI**	**Cramer's V**	***P*-value^#^**
Duration of marriage*			0.2 (IQR: 0.0–2.5)		
**Age gap**
No age gap	569,566 (16.2)	1,048 (11.7)	0.18 (0.17–0.20)	0.014	<0.001
Wife older than husband	555,002 (15.8)	1,482 (16.6)	0.27 (0.25–0.28)		
Husband 1–2 years older	1,069,184 (30.5)	2,332 (26.1)	0.22 (0.21–0.23)		
Husband 3–5 years older	923,330 (26.3)	2,336 (6.2)	0.25 (0.24–0.26)		
Husband ≥6 years older	393,230 (11.2)	1,723 (19.3)	0.44 (0.42–0.46)		
**Couple mobility**
Local couple	2,510,604 (71.5)	6,432 (72.1)	0.26 (0.25–0.26)	0.001	0.230
Migrant couple	999,708 (28.5)	2,489 (27.9)	0.25 (0.24–0.26)		
**Ever used condom**
No	2,642,464 (75.3)	7,354 (82.4)	0.28 (0.27–0.28)	0.008	<0.001
Yes	867,848 (24.7)	1,567 (17.6)	0.18 (0.17–0.19)		
**Number of children**
0	2,410,726 (68.7)	5,494 (61.6)	0.23 (0.22–0.23)	0.008	<0.001
≥1	1,099,586 (31.3)	3,427 (38.4)	0.31 (0.30–0.32)		
**Difference in education level**
Same education	2,409,532 (68.6)	6,176 (69.2)	0.26 (0.25–0.26)	0.001	0.486
Husband had higher education	580,632 (16.5)	1,446 (16.2)	0.25 (0.24–0.26)		
Wife had higher education	520,148 (14.8)	1,299 (14.6)	0.25 (0.24–0.26)		
**Region**
Pearl river delta	1,816,654 (51.8)	4,257 (47.7)	0.23 (0.23–0.24)	0.007	<0.001
East wing	401,444 (11.4)	1,321 (14.8)	0.33 (0.31–0.35)		
West wing	766,834 (21.8)	1,760 (19.7)	0.23 (0.22–0.24)		
Mountainous area	525,380 (15.0)	1,583 (17.7)	0.30 (0.29–0.32)		

Data was presented as No. (%); reported percentages are composition ratios of each horizontal item.

^#^The seroprevalence in subgroups was tested by Chi-square test.

*Data was described by median and interquartile range (IQR).

### Couple-level factors and syphilis infection

**Figure 3:**
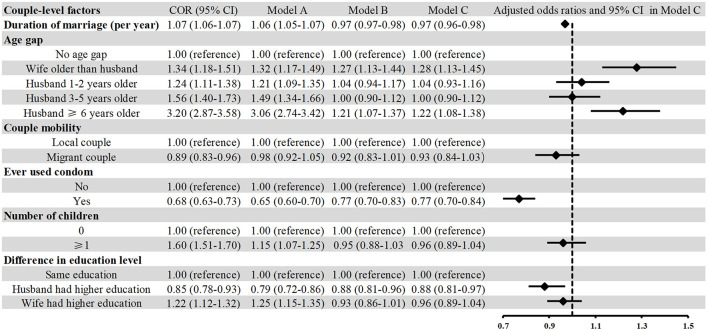
Association between couple-level factors and syphilis infection among men who participated in the National Free Preconception Health Examination Project in Guangdong Province, China during 2014–2019 (*N* = 1,755,156). Model A included only couple-level factors; Model B additionally adjusted for year of screening, age, ethnicity, educational level, household registration, smoking, drinking, migrant, illicit drug use, and previous STIs. Model C additionally adjusted for region.

**Figure 4:**
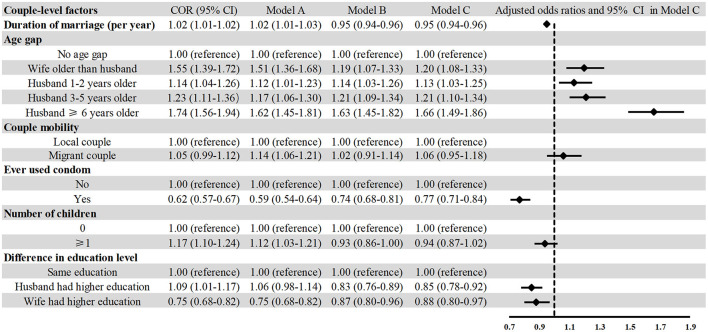
Association between couple-level factors and syphilis infection among women who participated in the National Free Preconception Health Examination Project in Guangdong Province, China during 2014–2019 (*N* = 1,755,156). Model A included only couple-level factors; Model B additionally adjusted for year of screening, age, ethnicity, educational level, household registration, smoking, drinking, migrant, illicit drug use, and previous STIs. Model C additionally adjusted for region.

As shown in Table 2, the seroprevalence in subgroups of couple mobility and difference in education level was not statistically significant (*P* = 0.230 and 0.486, respectively). Model C was adjusted for all individual-level and regional-level variables, including year of screening, age, ethnicity, educational level, household registration, smoking, drinking, migrant and region. Since syphilis infection was highly correlated between husbands and wives, we conducted general logistic regression stratified by gender. The results showed that duration of marriage was negatively associated with syphilis infection in men (AOR: 0.97; 95% CI: 0.96–0.98) and women (AOR: 0.95, 95% CI: 0.94–0.96) (Figures 3, 4). Compared to couples with no age difference, the wife being older than the husband was associated with a higher prevalence of syphilis in men (AOR: 1.28; 95% CI: 1.13–1.45). Any kind of age difference within the couple seemed to be positively associated with syphilis infection in women. Contrary to our hypothesis, couple-level mobility and the number of children were not statistically associated with syphilis infection in men and women. Condom use was negatively associated with syphilis infection in men (AOR: 0.77; 95% CI: 0.70–0.84) and women (AOR: 0.77; 95% CI: 0.71–0.84). Interestingly, higher education of either spouse or oneself was negatively associated with syphilis infection. However, having a spouse with a higher education for men was not statistically associated with syphilis infection (AOR: 0.96; 95% CI: 0.89–1.04).

Since the four regions of Guangdong Province are highly heterogeneous in terms of internal demographic composition, meteorological conditions, and economic conditions, we performed a stratified analysis to investigate whether the associations varied across regions. The results showed that for men, the associations between condom use, duration of marriage, and syphilis infection did not change across regions (Supplementary Table 3). The stratified analysis showed that, for women, having an older spouse was associated with a high risk of syphilis infection (Supplementary Table 4). As shown in Supplementary Table 5, the results were robust in sensitivity analysis for both men and women.

## Discussion

In this study, we explored couple-level determinants of syphilis infection among heterosexual married couples, after adjusting for individual-level and regional-level factors. We found that the duration of marriage, age gap, condom use, and difference in education level were all associated with syphilis infection in both men and women. Moreover, both couple mobility and the number of children were not statistically significant determinants of syphilis infection. This study helps us develop a more comprehensive picture of the way in which individuals' syphilis infection outcomes are situated in a larger relationship context.

We found that a longer duration of marriage was a protective factor for syphilis infection in men and women. It was consistent with a study of married couples in Thailand, which showed that a shorter duration of marriage (<2 years) was a risk factor for HIV transmission (33). However, a study in Northern Tanzania showed that a longer duration of marriage was a risk factor for HIV infection (17). The difference in study population and sample size may be the main reasons for these opposite results. Moreover, these studies were both hospital-based (17, 33), which could lead to selection bias. We used a population-based study to collect data from millions of couples, which could reduce bias and better evaluate disease epidemics (23).

According to the World Health Organization, sexual behavior between young women and older men was an important contributor to young people's vulnerability to HIV infection (34). However, two studies conducted in South Africa showed that the partner's age gap did not predict HIV acquisition among young women (34, 35). Moreover, few studies paid attention to the association between the age gap within couples and syphilis infection. Therefore, in this study, we explored this association and found that the age gap within couples was positively associated with syphilis infection. A population-based study in Botswana showed that intergenerational relationships (age gap ≥10 years) were positively associated with having multiple sexual partners and unprotected sex with non-primary partner (36). Consistent with a population-based study in rural China (22), we found that condom use was a protective factor for syphilis infection. However, 75.3% of couples in this study reported having never used condoms, which may reflect demonstrations of trust within this steady relationship (15). These results suggest that strategies to promote condom use among couples remain paramount to preventing syphilis (37).

We found that if the husband had a higher education level than his wife, it was a protective factor for syphilis infection for men. People with low educational levels might have less knowledge of syphilis (38) and also have limited access to preventive measures and treatment (39). A previous study reported that the partners' education does matter for women's maternal and reproductive health (40). However, little was known about the impact of partners' education on STIs. We found that for women, marrying someone with more education than themselves was a protective factor against syphilis infection. Partners' education may influence women's health outcomes through three main mechanisms (41). First, a well-educated partner may enhance the woman's economic status, a factor that plays an important role in health outcomes. Second, a woman could learn health information from her well-educated partner and also imitate his attitude toward health. Third, a well-educated partner may positively influence the woman to adopt healthier lifestyle choices and behaviors (40). But we found that for men, marrying someone with more education than themselves was not a statistically significant protective factor against syphilis infection. It was consistent with previous reports which suggested that spousal education was positively related to health, but to a greater degree for women than for men (18). Researchers have interpreted this phenomenon as a possible marker of a “resource substitution” process, meaning that because women have historically been less educated and less attached to the workforce, they place more value on the achievements of their spouses (18).

Migrants have been reported to be vulnerable to STIs (42). However, studies on the relationship between couple-level mobility and syphilis infection are scarce (20). In the present study, we classified couples as local couples (the wife and husband were both native residents) or migrant couples (either the wife or husband or both were migrant). We found that couple-level mobility was not statistically associated with syphilis infection. This may be due to the target populations, as this study was limited to married migrants, who are at lower risk of having multiple sexual partners (36). Our finding that the number of children was not statistically associated with syphilis infection runs counter to a national study in China, which showed that previous pregnancy was a risk factor for syphilis infection (22). This could be partly explained by the different classification criteria, as the number of children rather than previous pregnancy was used as an independent variable in this study.

To the best of our knowledge, this study was the first to explore couple-level determinants of syphilis infection among married couples. This study has several strengths. First, the NFPHEP was well-established, had standard quality control, and the data obtained by trained personnel was reliable. Second, this study included an extremely large dataset, which enabled us to explore the couple-level determinants of this relatively low-incidence disease. Third, this study investigated the association of couple-level factors with syphilis infection in men and women separately. However, this study also has several limitations. First, this study was a cross-sectional study, which limits our ability to make causal inference. Therefore, it must be noted that the associations in this study should be understood in a statistical rather than a causal relationship sense. Second, recall bias might exist as some independent factors were self-reported (e.g., educational level, number of children, and so on). Third, only the operational data was used in the analyses. Therefore, we cannot entirely rule out the potential residual confounding caused by unmeasured or unknown factors. Further improvement could be made when the information can be obtained. Fourth, only couples planning a pregnancy were included in this study, which may have led to selection bias. Further studies are needed to conduct in the general population. However, we believe that our main findings carry important implications for prevention efforts and further research.

This study contributes to a more comprehensive understanding of syphilis outcomes in individuals in the context of marriage in China. The results of this study suggest that couple-level factors are indeed associated with syphilis infection, and these associations differ between men and women. Couple-based strategies that engage both women and men and efforts to promote condom use among heterosexual married couples need to be developed and further evaluated for syphilis prevention.

## Data availability statement

The datasets presented in this article are not readily available because privacy and ethical issue. Access to these data may be requested through the Guangdong Provincial Reproductive Science Institute for researchers who meet the criteria for access to this syphilis data. Requests to access the datasets should be directed to LH, sysuhanlu@126.com.

## Ethics statement

The studies involving human participants were reviewed and approved by Institutional Review Board of the Chinese Association of Maternal and Child Health Studies. The patients/participants provided their written informed consent to participate in this study.

## Author contributions

LH, WX, LL, and XL were involved in the study conceptualization and study design. WX, ML, and RL carried out statistical analyses. The first draft of the article was written by LH and WX. ML, JW, XT, LL, and XL provided critical revision of the article for important intellectual content. All authors contributed to the interpretation of the data and approved the final version of the article.

## Funding

This work was supported by the National Natural Science Foundation of China grants (No. 8210050633), the Natural Science Foundation of Guangdong Province (Nos. 2019A1515011984 and 2020A1515110735), and Guangdong Province Medical Research Funding (Nos. 2022314 and 2022326).

## Conflict of interest

The authors declare that the research was conducted in the absence of any commercial or financial relationships that could be construed as a potential conflict of interest.

## Publisher's note

All claims expressed in this article are solely those of the authors and do not necessarily represent those of their affiliated organizations, or those of the publisher, the editors and the reviewers. Any product that may be evaluated in this article, or claim that may be made by its manufacturer, is not guaranteed or endorsed by the publisher.
