# Supplementary material for: Couple-level determinants of syphilis infection among heterosexual married couples of reproductive age in Guangdong Province, China: A population-based cross-sectional study
Source: Front Public Health. 2022 Oct 17;10:1004246. doi: 10.3389/fpubh.2022.1004246 (PMC9620861; doi:10.3389/fpubh.2022.1004246)
Supplement: Supplementary file 1 [file Data_Sheet_1.docx]

**Supplementary Table 1. Description of the individual-level, couple-level, and regional-level factors.**

| **Factors** | **Description** |
| --- | --- |
| **Individual-level** | Individual-level factors were obtained by trained health workers using a standardized questionnaire. |
| Outcome | The result of the RPR test (positive or negative). |
| Year of screening | Year of participation in the project (2014, 2015, 2016, 2017, 2018 or 2019). |
| Gender | Self-reported (man or women). |
| Age | Self-reported (continuous variable). |
| Ethnicity | Self-reported (Han or minority). |
| Educational level | Self-reported (primary school or below, middle and high school or college and above). |
| Household registration | Based on the HUKOU system (rural or urban). |
| Smoking | *Do you smoke?* Self-reported (yes or no). |
| Drinking | *Do you drink?* Self-reported (yes or no). |
| Migrant | Whether the municipality where they participated in the project is different from the one where they were registered (yes or no). |
| Previous STIs | Presence of any of the following four sexually transmitted infections: gonorrhea, syphilis, chlamydia, and/or hepatitis B. Self-reported (yes or no). |
| Illicit drug use | *Do you use illegal drugs?* Self-reported (yes or no). |
| **Couple-level** | Couple-level factors were obtained by combining information provided by the man and woman living in the same household. |
| Duration of marriage | The difference in time (in years) between participation in the NFPHCP and registration of the marriage (continuous variable). |
| Age gap | The age difference between the husband and wife (no age gap, wife older than husband, husband 1-2 years older, husband 3-5 years older, or husband ≥ 6 years older). |
| Couple mobility | Local couples (both the wife and husband were native residents) or migrant couples (either the wife or husband or both were migrant). |
| Ever used condom | *Have you ever used the condom for contraception?* Self-reported (yes or no). |
| Number of children | Self-reported (0 or ≥1). |
| Difference in education level | Difference in education level between the husband and wife (same education level, husband had a higher education, or wife had a higher education). |
| **Regional-level** |  |
| Region | Guangdong Province can be divided into four regions: Pearl River Delta (Guangzhou, Shenzhen, Zhuhai, Foshan, Huizhou, Dongguan, Zhongshan, Jiangmen and Zhaoqing), East Wing (Shantou, Shanwei, Chaozhou and Jieyang), West Wing (Yangjiang, Zhanjiang and Maoming), or Mountainous Area (Shaoguan, Heyuan, Meizhou, Qingyuan and Yunfu). |

**Supplementary Table 2. Basic characteristics of married individuals who participated in** **National Free Preconception Health Examination Project in Guangdong Province, China during 2014-2019, stratified by gender.**

| **Characteristic** | **Men (N=1,751,156)** | | ***P^#^*** | **Women(N=1,751,156)** | | ***P*^#^** |
| --- | --- | --- | --- | --- | --- | --- |
|  | **Total no. (%)** | **Cases (%)** |  | **Total no. (%)** | **Cases (%)** |  |
| **Individual-level factors** |  |  |  |  |  |  |
| **Year of screening** |  |  | < 0.001 |  |  | 0.666 |
| 2014 | 325,860 (18.6) | 906 (20.8) |  | 325,860 (18.6) | 876 (19.2) |  |
| 2015 | 290,144 (16.5) | 741 (17.0) |  | 290,144 (16.5) | 723 (15.8) |  |
| 2016 | 350,134 (19.9) | 1,047 (24.1) |  | 350,134 (19.9) | 921 (20.1) |  |
| 2017 | 312,820 (17.8) | 712 (16.4) |  | 312,820 (17.8) | 833 (18.2) |  |
| 2018 | 270,330 (15.4) | 553 (12.7) |  | 270,330 (15.4) | 689 (15.1) |  |
| 2019 | 205,868 (11.7) | 391 (9.0) |  | 205,868 (11.7) | 529 (11.6) |  |
| **Age (year)*** | 29.0 (26.0-32.0) | |  | 27.0 (24.0-30.0) | |  |
| **Ethnicity^##^** |  |  | 0.590 |  |  | < 0.001 |
| Han | 1,738,514 (99.2) | 4,305 (99.1) |  | 1,735,176 (99.1) | 4,488 (98.3) |  |
| Minority | 11,478 (0.8) | 37 (0.9) |  | 16,414 (0.9) | 77 (1.7) |  |
| **Educational level** |  |  | < 0.001 |  |  | < 0.001 |
| Primary school or below | 35,669 (2.0) | 186 (4.3) |  | 40,049 (2.3) | 219 (4.8) |  |
| Middle and high school | 1,029,363 (58.6) | 3,118 (71.7) |  | 1,015,031 (57.8) | 3,319 (72.6) |  |
| College and above | 690,124 (39.3) | 1,046 (24.0) |  | 700,076 (39.9) | 1,033 (22.6) |  |
| **Household registration^##^** |  |  | <0.001 |  |  | < 0.001 |
| Rural | 1,246,021 (71.1) | 3,259 (74.9) |  | 1,295,154 (73.9) | 3,659 (80.1) |  |
| Urban | 507,203 (28.9) | 1,090 (25.1) |  | 458,383 (26.1) | 908 (19.9) |  |
| **Smoking^##^** |  |  | <0.001 |  |  | < 0.001 |
| No | 1,269,782 (72.6) | 2,670 (61.7) |  | 1,741,969 (99.6) | 4,489 (98.6) |  |
| Yes | 478,318 (27.4) | 1,660 (38.3) |  | 6,658 (0.4) | 64 (1.4) |  |
| **Drinking^##^** |  |  | < 0.001 |  |  | 0.013 |
| No | 1,104,977 (63.2) | 2,627 (60.7) |  | 1,594,566 (91.3) | 4,106 (90.3) |  |
| Yes | 642,771 (36.8) | 1,703 (39.3) |  | 151,815 (8.7) | 443 (9.7) |  |
| **Migrant** |  |  | 0.661 |  |  | 0.002 |
| No | 1,476,779 (84.1) | 3,649 (83.9) |  | 1,416,330 (80.7) | 3,607 (78.9) |  |
| Yes | 278,377 (15.9) | 701 (16.1) |  | 338,826 (19.3) | 964 (21.1) |  |
| **Previous STI** |  |  | <0.001 |  |  | < 0.001 |
| No | 1,719,490 (98.0) | 4,163 (95.7) |  | 1,724,735 (98.3) | 4,349 (95.1) |  |
| Yes | 35,666 (2.0) | 187 (4.3) |  | 30,421 (1.7) | 222 (4.9) |  |
| **Drug use^##^** |  |  | <0.001 |  |  | <0.001 |
| No | 1,742,909 (100.0) | 4,317 (99.9) |  | 1,743,957 (100.0) | 4,529 (99.9) |  |
| Yes | 389 (0.0) | 4 (0.1) |  | 132 (0.0) | 3 (0.1) |  |
| **Couple-level factors** |  |  |  |  |  |  |
| **Duration of marriage (year)*** | 0.1（0.0-2.4） | |  | 0.1（0.0-2.4） | |  |
| **Age gap** |  |  | <0.001 |  |  | < 0.001 |
| No age gap | 284,783 (16.2) | 465 (10.7) |  | 284,783 (16.2) | 583 (12.8) |  |
| Wife older than husband | 277,501 (15.8) | 605 (13.9) |  | 277,501 (15.8) | 877 (19.2) |  |
| Husband 1-2 years older | 534,592 (30.5) | 1,082 (24.9) |  | 534,592 (30.5) | 1,250 (27.3) |  |
| Husband 3-5 years older | 461,665 (26.3) | 1,173 (27.0) |  | 461,665 (26.3) | 1,163 (25.4) |  |
| Husband ≥ 6 years older | 196,615 (11.2) | 1,025 (23.6) |  | 196,615 (11.2) | 698 (15.3) |  |
| **Couple mobility** |  |  | 0.001 |  |  | 0.134 |
| Local couple | 1,255,302 (71.5) | 3,209 (73.8) |  | 1,255,302 (71.5) | 3,223 (70.5) |  |
| Migrant couple | 499,854 (28.5) | 1,141 (29.5) |  | 499,854 (28.5) | 1,348 (29.5) |  |
| **Ever used condom** |  |  | < 0.001 |  |  | < 0.001 |
| No | 1,321,232 (75.3) | 3,555 (81.7) |  | 1,321,232 (75.3) | 3,799 (83.1) |  |
| Yes | 433,924 (24.7) | 795 (18.3) |  | 431,924 (24.7) | 772 (16.9) |  |
| **Number of children** |  |  | < 0.001 |  |  | < 0.001 |
| 0 | 1,205,363 (68.7) | 2,514(57.8) |  | 1205363 (68.7) | 2,980 (65.2) |  |
| ≥1 | 549,793 (31.3) | 1,836 (42.2) |  | 549793 (31.3) | 1,591 (34.8) |  |
| **Difference in education level** |  |  | < 0.001 |  |  | < 0.001 |
| Same education | 1,204,766 (68.6) | 2,966 (68.2) |  | 1204766 (68.6) | 3,210 (70.2) |  |
| Husband had higher education | 290,316 (16.5) | 606 (13.9) |  | 290316 (16.5) | 840 (18.4) |  |
| Wife had higher education | 260,074 (14.8) | 778 (17.9) |  | 260074 (14.8) | 521 (11.4) |  |
| **Region** |  |  | <0.001 |  |  | <0.001 |
| Pearl River Delta | 908,327 (51.8) | 2,205 (50.7) |  | 901,327 (51.8) | 2,052 (44.9) |  |
| East Wing | 200,722 (11.4) | 570 (13.1) |  | 201,722 (11.4) | 751 (16.4) |  |
| West Wing | 382,417 (21.8) | 815 (18.7) |  | 381,417 (21.8) | 945 (20.7) |  |
| Mountainous Area | 262,690 (15.0) | 760 (17.5) |  | 261,690 (15.0) | 823 (18.0) |  |

Data was presented as No. (%); reported percentages are composition ratios of each horizontal item.

^#^ The seroprevalence in subgroups was tested by Chi-square test.

* Data was described by median and interquartile range (IQR).

^##^ 3,566 (0.2%) wives and 3,164 (0.2%) husbands missing in ethnicity; 1,619 (0.1%) wives and 1,932 (0.1%) husbands missing in household registration; 6,529 (0.4%) wives and 7,056 (0.4%) husbands missing in smoking; 8,775 (0.5%) wives and 7,408 (0.4%) husbands missing in drinking; 11,968 (0.7%) wives and 11,858 (0.7%) husbands missing in drug use.

**Supplementary Table 3.** **Association between couple-level factors and syphilis infection among men who participated in the National Free Preconception Health Examination Project in Guangdong during 2014-2019, stratified by region (N= 1,755,156).** Adjusted for year of screening, age, ethnicity, educational level, household registration, smoking, drinking, migrant, illicit drug use, and previous STIs. The bold numbers indicated *P* < 0.05.

| **Couple-level factors** | **Pearl River Delta** | **East Wing** | **West Wing** | **Mountainous Area** |
| --- | --- | --- | --- | --- |
| **Duration of marriage (per year)** | **0.98 (0.97-0.99)** | **0.90 (0.84-0.97)** | **0.97 (0.94-1.00)** | **0.94 (0.93-0.96)** |
| **Age gap** |  |  |  |  |
| No age gap | 1.00 (reference) | 1.00 (reference) | 1.00 (reference) | 1.00 (reference) |
| Wife older than husband | **1.60 (1.35-1.90)** | 0.90 (0.64-1.27) | 1.00 (0.75-1.34) | 1.09 (0.82-1.47) |
| Husband 1-2 years older | 1.14 (0.97-1.33) | 1.02 (0.78-1.33) | 0.92 (0.71-1.18) | 0.94 (0.72-1.22) |
| Husband 3-5 years older | 1.17 (1.00-1.37) | 0.89 (0.67-1.18) | 0.87 (0.68-1.12) | 0.83 (0.64-1.08) |
| Husband ≥ 6 years older | **1.31 (1.09-1.56)** | **1.63 (1.16-2.30)** | 1.05 (0.80-1.40) | 1.03 (0.77-1.36) |
| **Couple mobility** |  |  |  |  |
| Local couple | 1.00 (reference) | 1.00 (reference) | 1.00 (reference) | 1.00 (reference) |
| Migrant couple | 0.90 (0.78-1.03) | 1.23 (0.89-1.71) | **0.74 (0.56-0.97)** | 1.15 (0.92-1.45) |
| **Ever used condom** |  |  |  |  |
| No | 1.00 (reference) | 1.00 (reference) | 1.00 (reference) | 1.00 (reference) |
| Yes | **0.79 (0.72-0.87)** | 1.12 (0.70-1.79) | **0.46 (0.27-0.77)** | **0.68 (0.52-0.89)** |
| **Number of children** |  |  |  |  |
| 0 | 1.00 (reference) | 1.00 (reference) | 1.00 (reference) | 1.00 (reference) |
| ≥1 | 0.93 (0.83-1.05) | 1.08 (0.84-1.38) | 0.88 (0.74-1.05) | 1.09 (0.92-1.30) |
| **Difference in education level** |  |  |  |  |
| Same education | 1.00 (reference) | 1.00 (reference) | 1.00 (reference) | 1.00 (reference) |
| Husband had higher education | 0.96 (0.85-1.08) | **0.64 (0.49-0.85)** | 0.89 (0.73-1.09) | 0.84 (0.68-1.05) |
| Wife had higher education | 1.06 (0.94-1.18) | **0.60 (0.44-0.82)** | **0.81 (0.66-0.99)** | 1.03 (0.84-1.26) |

**Supplementary Table 4.** **Association between couple-level factors and syphilis infection among women who participated in the National Free Preconception Health Examination Project in Guangdong during 2014-2019, stratified by region (N= 1,755,156).** Adjusted for year of screening, age, ethnicity, educational level, household registration, smoking, drinking migrant, illicit drug use, and previous STIs. The bold numbers indicated *P* < 0.05.

| **Couple-level factors** | **Pearl River Delta** | **East Wing** | **West Wing** | **Mountainous Area** |
| --- | --- | --- | --- | --- |
| **Duration of marriage (per year)** | **0.96 (0.95-0.98)** | 0.98 (0.93-1.04) | **0.94 (0.91-0.96)** | **0.93 (0.91-0.95)** |
| **Age gap** |  |  |  |  |
| No age gap | 1.00 (reference) | 1.00 (reference) | 1.00 (reference) | 1.00 (reference) |
| Wife older than husband | 1.09 (0.93-1.26) | **1.52 (1.14-2.01)** | **1.26 (0.99-1.61)** | 1.14 (0.89-1.46) |
| Husband 1-2 years older | 1.08 (0.93-1.25) | **1.29 (1.01-1.65)** | 1.10 (0.88-1.38) | 1.11 (0.88-1.40) |
| Husband 3-5 years older | **1.24 (1.07-1.44)** | 1.22 (0.95-1.58) | 1.23 (0.98-1.55) | 1.05 (0.83-1.35) |
| Husband ≥ 6 years older | **1.69 (1.44-1.99)** | **1.52 (1.11-2.08)** | **1.61 (1.25-2.07)** | **1.66 (1.29-2.16)** |
| **Couple mobility** |  |  |  |  |
| Local couple | 1.00 (reference) | 1.00 (reference) | 1.00 (reference) | 1.00 (reference) |
| Migrant couple | 1.06 (0.92-1.22) | 1.03 (0.70-1.51) | 0.90 (0.66-1.22) | 1.22 (0.93-1.60) |
| **Ever used condom** |  |  |  |  |
| No | 1.00 (reference) | 1.00 (reference) | 1.00 (reference) | 1.00 (reference) |
| Yes | **0.75 (0.68-0.83)** | 0.61 (0.37-1.01) | 0.99 (0.71-1.37) | 0.97 (0.77-1.23) |
| **Number of children** |  |  |  |  |
| 0 | 1.00 (reference) | 1.00 (reference) | 1.00 (reference) | 1.00 (reference) |
| ≥1 | **0.76 (0.67-0.87)** | 1.15 (0.93-1.42) | 1.07 (0.91-1.25) | 0.98 (0.83-1.16) |
| **Difference in education level** |  |  |  |  |
| Same education | 1.00 (reference) | 1.00 (reference) | 1.00 (reference) | 1.00 (reference) |
| Husband had higher education | 0.90 (0.80-1.01) | **0.68 (0.54-0.85)** | **0.84 (0.71-1.00)** | 0.89 (0.73-1.08) |
| Wife had higher education | 0.96 (0.84-1.10) | 0.80 (0.61-1.05) | 0.87 (0.71-1.06) | **0.79 (0.62-1.00)** |

**Supplementary Table 5. Results of sensitivity analysis.** In the sensitivity analysis, a “Not available” category for those with missing data at individual-level was created. Adjusted for year of screening, age, ethnicity, educational level, household registration, smoking, drinking, migrant, illicit drug use, and previous STIs. The bold numbers indicated *P* < 0.05.

| **Couple-level factors** | **Men** | **Women** |
| --- | --- | --- |
| **Duration of marriage (per year)** | **0.97 (0.96-0.98)** | **0.95 (0.94-0.96)** |
| **Age gap** |  |  |
| No age gap | 1.00 (reference) | 1.00 (reference) |
| Wife older than husband | **1.29 (1.15-1.46)** | **1.20 (1.08-1.34)** |
| Husband 1-2 years older | 1.05 (0.94-1.17) | **1.12 (1.02-1.24)** |
| Husband 3-5 years older | 1.01 (0.91-1.13) | **1.21 (1.10-1.34)** |
| Husband ≥ 6 years older | **1.22 (1.08-1.37)** | **1.67 (1.50-1.87)** |
| **Couple mobility** |  |  |
| Local couple | 1.00 (reference) | 1.00 (reference) |
| Migrant couple | 0.93 (0.84-1.03) | 1.06 (0.95-1.18) |
| **Ever used condom** |  |  |
| No | 1.00 (reference) | 1.00 (reference) |
| Yes | **0.77 (0.71-0.84)** | **0.77 (0.71-0.84)** |
| **Number of children** |  |  |
| 0 | 1.00 (reference) | 1.00 (reference) |
| ≥1 | 0.97 (0.89-1.05) | 0.94 (0.87-1.02) |
| **Difference in education level** |  |  |
| Same education | 1.00 (reference) | 1.00 (reference) |
| Husband had higher education | **0.88 (0.80-0.96)** | **0.85 (0.78-0.92)** |
| Wife had higher education | 0.95 (0.87-1.03) | **0.88 (0.80-0.97)** |
